# Supplementary material for: Wide-field mid-infrared hyperspectral imaging beyond video rate
Source: Nat Commun. 2024 Feb 28;15:1811. doi: 10.1038/s41467-024-46274-z (PMC10902379; doi:10.1038/s41467-024-46274-z)
Supplement: Supplementary file 1 — Supplementary Information [file 41467_2024_46274_MOESM1_ESM.pdf]

Wide-field mid-infrared hyperspectral imaging beyond video rate
# - Supplementary Information -

Jianan Fang,[1] Kun Huang,[1,2,3,*] Ruiyang Qin,[1] Yan Liang,[4] E Wu,[1,2] Ming Yan,[1,2] and Heping Zeng[1,2,5,6,†]

[1] *State Key Laboratory of Precision Spectroscopy, East China Normal University, Shanghai 200062, China*
[2] *Chongqing Key Laboratory of Precision Optics, Chongqing Institute of East China Normal University, Chongqing 401121, China*
[3] *Collaborative Innovation Center of Extreme Optics, Shanxi University, Taiyuan, Shanxi 030006, China*
[4] *School of Optical Electrical and Computer Engineering, University of Shanghai for Science and Technology, Shanghai 200093, China*
[5] *Shanghai Research Center for Quantum Sciences, Shanghai 201315, China*
[6] *Chongqing Institute for Brain and Intelligence, Guangyang Bay Laboratory, Chongqing, 400064, China*

Supplementary Note 1: Theoretical model for upconversion imaging

The mid-infrared (MIR) spectral upconversion imaging in our experiment is implemented based on a 4-f configuration, as schematically illustrated in Fig. S1(A). The nonlinear process relies on sum-frequency generation (SFG) within a chirped-poling lithium niobate (CPLN) crystal. The involved optical fields satisfy the energy conservation law as $1/\lambda_u = 1/\lambda_s + 1/\lambda_p$, where $\lambda_{s,p,u}$ are wavelengths in vacuum for the signal, pump and upconverted light. Moreover, the momentum conservation should be fulfilled to approach an efficient frequency conversion. The phase-mismatching vector can be decomposed into the axial and transverse directions as

$$\frac{\Delta k_{\parallel}}{2\pi} = \frac{n_u}{\lambda_u}\cos\theta_u - \frac{n_s}{\lambda_s}\cos\theta_s - \frac{n_p}{\lambda_p} - \frac{1}{\Lambda} \ , \tag{1}$$

$$\frac{\Delta k_{\perp}}{2\pi} = \frac{n_u}{\lambda_u}\sin\theta_u - \frac{n_s}{\lambda_s}\sin\theta_s \ , \tag{2}$$

where $n_{s,p,u}$ are the refractive indices, and $\theta_{s,p,u}$ denote the beam angles within the crystal. For a given incident angle θ_s for the infrared signal, it is possible to optimize the angle θ_u for the upconverted beam with an adapted poling period Λ under the phase-matching restriction of $|\Delta\vec{k}| = \sqrt{\Delta k_{\parallel}^2 + \Delta k_{\perp}^2} = 0$ [1]. Consequently, the phase-matching condition can be realized for the MIR signal with different incident angles over a broadband spectrum, which contrasts to the narrow-band performance based on periodically poled lithium niobate (PPLN) crystals [2, 3]. The resulting broadband and wide-field operation constitutes the core to implement a single-shot conversion of the MIR spectral imaging, which excludes the need for parameter scanning or post-processing [4].

*Electronic address: khuang@lps.ecnu.edu.cn
†Electronic address: hpzeng@phy.ecnu.edu.cn

Particularly, an angular scaling factor for the external angles $\theta'_{s,u}$ can be deduced from the transverse momentum conservation according to Eq. (2):

$$\frac{\sin\theta'_u}{\sin\theta'_s} = \frac{\lambda_u}{\lambda_s}\ , \tag{3}$$

where we use the Snell's law for the angles inside and outside the crystal: $\sin\theta'_{s,u} = n_{s,u} \times \sin\theta_{s,u}$. In the presence of two relay lens f_1 and f_2 in the 4-f imaging system, the corresponding spatial scaling factor is given by

$$M(\lambda_s, \theta'_s) = \frac{f_2 \times \tan\theta'_u}{f_1 \times \tan\theta'_s} = \frac{f_2\lambda_u}{f_1\lambda_s} \times \sqrt{\frac{1-\sin^2\theta'_s}{1-\sin^2\theta'_s \times \lambda_u^2/\lambda_s^2}}\ . \tag{4}$$

Under the small-angle approximation, the above formula can be simplified to

$$M(\lambda_s) = \frac{f_2}{f_1} \times \frac{\lambda_u}{\lambda_s} = \frac{f_2}{f_1} \times \frac{1}{\lambda_s/\lambda_p + 1}\ , \tag{5}$$

which depends on the wavelength ratio between the signal and pump beams.

Figure S1(B) presents the theoretical simulation for the spatial scaling factor depending on the external input angle and the infrared signal wavelength. At a given wavelength, the scaling factor

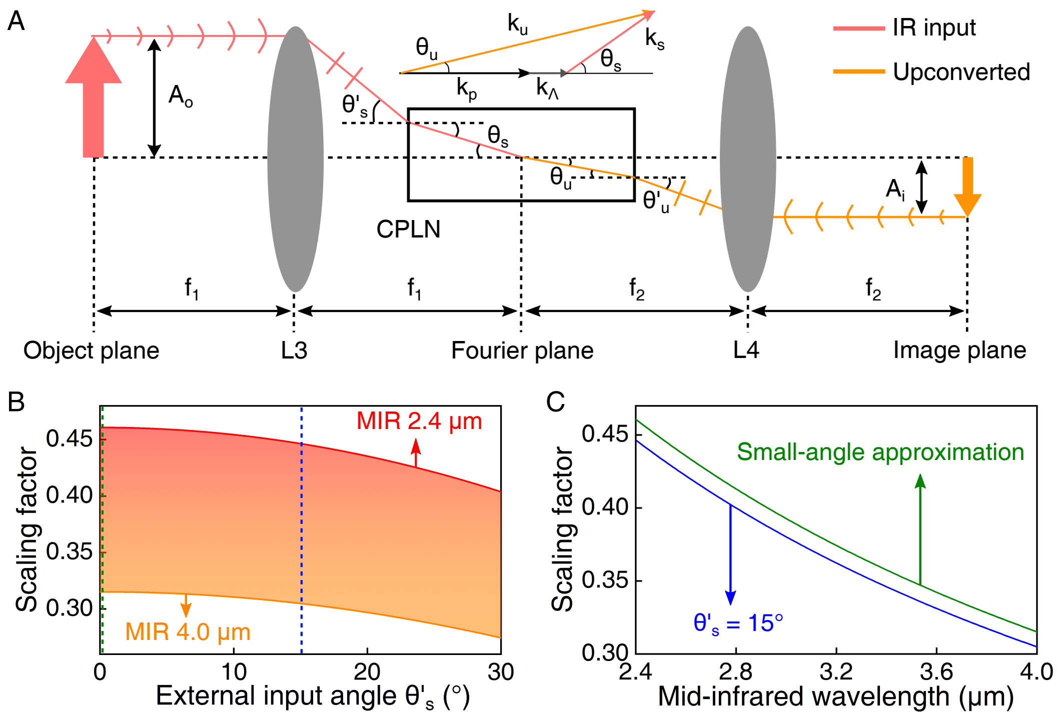

Figure S1: Theoretical model and simulation for the wavelength-dependent spatial scaling during the frequency upconversion. (A) Schematic illustration of the parametric upconversion imaging in a 4-f architecture. (B) Spatial scaling factor as a function of the external input angle of the infrared signal over a broadband spectrum from 2.4 to 4 μm. The vertical dashed line indicates the acceptance angle in our experiment. (C) Scaling factors vary as the increase of the MIR wavelength in the presence of small incident angles and an angle of 15°, which are evaluated by the simplified and rigorous models, respectively.

becomes smaller as the increase of the incident angle. In the object plane, the magnification of the image decreases as the object is farther from the optical axis of the lens, which results in an image deformation similar to the barrel lens distortion. In our experiment, the acceptance angle of the upconversion imaging system is about 15°, which is determined by the one-inch optical lens. Within the acceptance angle, the scaling variation is about 3.1% and 3.3% at 2.4 and 4 μm, respectively. In the polychromatic operation, the scaling factor increases at a shorter signal wavelength, which leads to a blurring effect in the upconverted spectral images. Specifically, the wavelength-dependent variation of the spatial scaling is investigated in Fig. S1(C) based on the simplified and rigorous models for small incident angles and an angle of 15°, respectively. The relative deviation for the simplified model is below 1% for input angles smaller than 8°. In our experiment, the scaling map is precisely characterized in prior based on the rigorous model. The rescaling operation for each monochromatic image can be fast computed via simple matrix manipulations.

Supplementary Note 2: Details about experimental setup

The detailed schematic for the experimental setup is presented in Fig. S2, which is comprised of three main parts: synchronous pulse preparation, broadband frequency upconversion, and high-speed hyperspectral imaging. The involved signal light source originates from a MIR supercontinuum (SC) fiber laser (Novae, Coverage) with a spectral coverage of 1.9-3.9 μm. The laser delivers a train of ultrashort pulses with a duration of 40 ps at a repetition frequency of 2.35 MHz. The output beam is divided into two portions by a 2.4 μm long-pass filter (Edmund, #33-969). The transmitted beam passes through a half-wave plate (Thorlabs, WPLH05M-2940) and a polarizer (Thorlabs, WP25M-IRA), allowing us to adjust the illumination power and to ensure a vertical polarization state. After the spectral and polarization filtering, the maximum illumination power on the sample is about 113 mW. Then, the MIR beam is expanded to approximately 4 cm in diameter through a beam expander, which results in a spectral intensity is about 7 μW/nm/cm^2. Meanwhile, the reflective portion is recorded by an InGaAs detector (Thorlabs, PDA20CS2), which serves an external reference for an electrical pulse generator (Agilent, 81130A) to generate a synchronous square wave signal. The square wave is used to modulate a laser diode (LD-PD Inc., PL-DFB-1064) at 1064 nm. The pulse duration is set to be 200 ps, which is sufficient to enwrap the MIR pulse. The timing delay can be electronically controlled with a precision of 1 ps, which is essential to optimize the overlap between the dual-color pulses in the subsequent upconversion imaging. The average power is boosted to 600 mW by using two-stage fiber amplifiers (YDFA). The corresponding spectral bandwidth is about 0.2 nm, which ensures a high-fidelity spectral mapping during the nonlinear conversion.

The MIR illumination after a sample is steered into a 4-f imaging system consisting of two relay lenses with focal lengths of f_1 = 50 mm and f_2 = 75 mm. At the Fourier plane, a CPLN nonlinear crystal is used to perform the SFG process. The crystal size is 3×2×10 mm^3 (width×thickness×length). The poling period linearly ramps from 16 to 24 μm along the propagation direction, which allows for a wide acceptance angle up to 20° over a spectral window of 2.4-5 μm. The wide-field and broadband nonlinear conversion constitutes the key to realizing the spec-

tral imaging mapping in a single shot. The upconverted beam is sent into an acousto-optic tunable filter (AOTF) in order to extract monochromatic images. The AOTF features with a high-speed and inertia-free spectral filtering. Detailed characterization of the temporal and spectral responses will be discussed in the next section. The filtered beam in first-order refraction is deflected into a silicon-based CMOS camera (Photron, Mini AX200), while the zero-order beam is blocked by a beam stop. The camera sensor is equipped with 1024×1024 pixels, which permits a refreshing rate to 6.4 kHz at the full frame. Higher frame rate can be obtained with a reduced number of active pixels. For instance, frames with 768×768 and 512×512 pixels can be recorded at maximum acquisition rates of 10.8 kHz and 22.5 kHz, respectively. The available frame rates are at least two orders of magnitude faster than the state-of-the-art values for MIR focal plane arrays (FPAs). It is the simultaneous realization of fast wide-field detection and fast spectral filtration that enables us to boost the acquisition speed for the hyperspectral images. To suppress background noise from

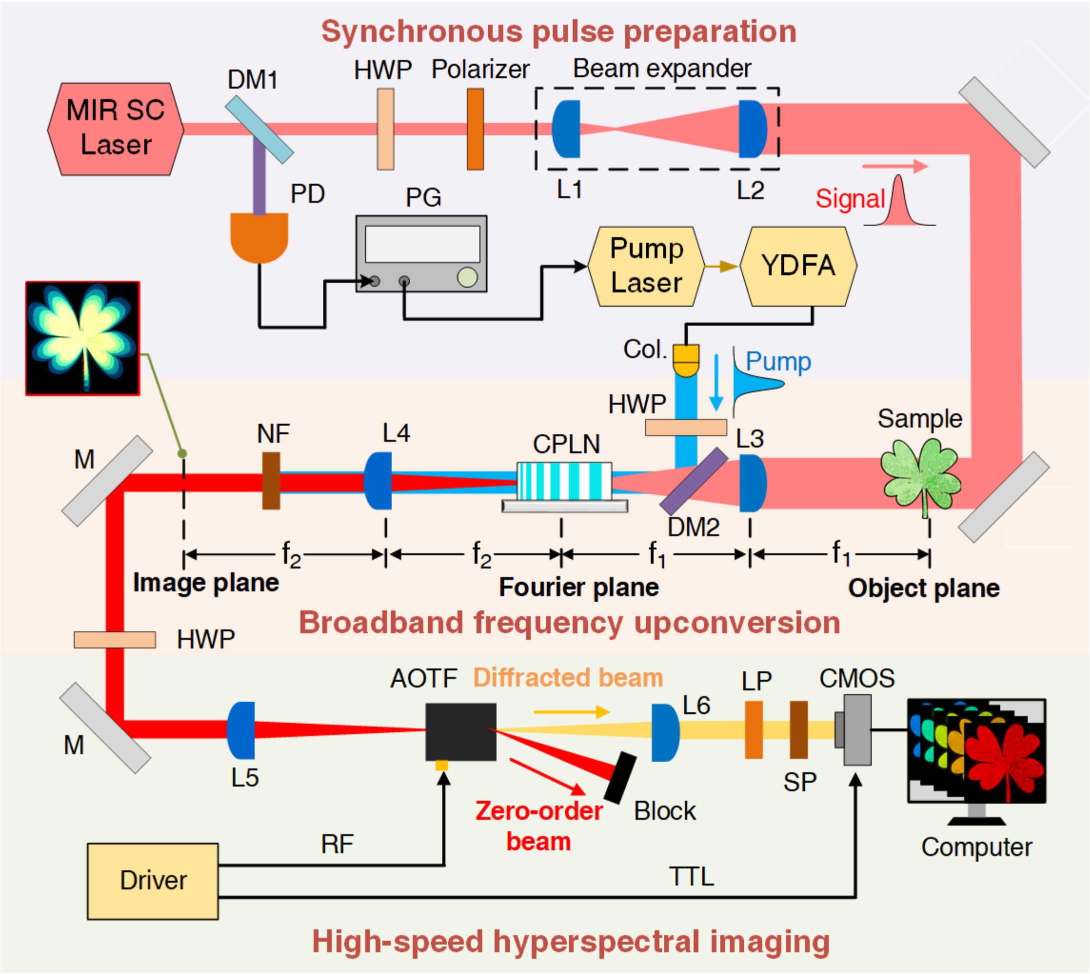

Figure S2: Schematic for the experimental setup, which mainly consists of three parts for synchronous pulse preparation, broadband frequency upconversion, and high-speed hyperspectral imaging. CPLN: chirped-poling niobate crystal; YDFA: Yb-doped fiber amplifier; Col.: collimator; DM: dichroic mirror; L: lens; M: silver mirror; HWP: half-wave plate; PD: photodiode; PG: pulse generator; AOTF: acousto-optic tunable filter; NF: notch filter; LP: long-pass filter; SP: short-pass filter.

the pump-induced fluorescence, the filtered beam passes through a series of interference filters, including a notch filer (Thorlabs, NF1064-44), a short-pass filter (Edmund Optics, #64335), and a long-pass filter (Edmund Optics, #68653). The total transmission is measured to be about 80%, and the rejection ratio at the pump wavelength is estimated to be over 180 dB.

Supplementary Note 3: Operating configuration for acquisition timing

The realization of the MIR hyperspectral imaging needs to synchronize the spectral filter and imaging camera, which is particularly important in the high-speed scenario. In our experiment, the involved timing for device synchronization is managed by a programmed driver based on a field-programmable gate array (FPGA). As shown in Fig. S3, the driver outputs a sequence of square wave as the trigger signal for the AOTF and camera. The AOTF is switched at the rising edge, and will be stabilized within several μs. The camera starts to capture images after a temporal delay t_1, and has an exposure time t_2. The total acquisition time for N spectral channels is thus $N \times T$, where T is the period for each monochromatic operation state. Notably, there is no dead time between two sequential cycles due to the inertial-free filtering feature of the AOTF, which allows us to implement a real-time spectral imaging visualization over a long acquisition period. Notably, the spectral channels are not required to be evenly located within the interrogated spectrum. The wavelength points can be flexibly programmed while maintaining the total acquisition time, which is useful to conduct an efficient sampling over certain featured spectral regions.

To characterize the spectral and temporal responses of the AOTF, a super-luminescent light emitting diode laser (SLED) is used to provide a continuous-wave broadband light source. The filtering central wavelength decreases linearly as the increase of the driving frequency of the RF signal, as shown in Fig. S4(A). Generally, the AOTF performances are determined by the intrinsic property and geometry size of the acousto-optic crystal [5]. As given in Fig. S4(B), the spectral

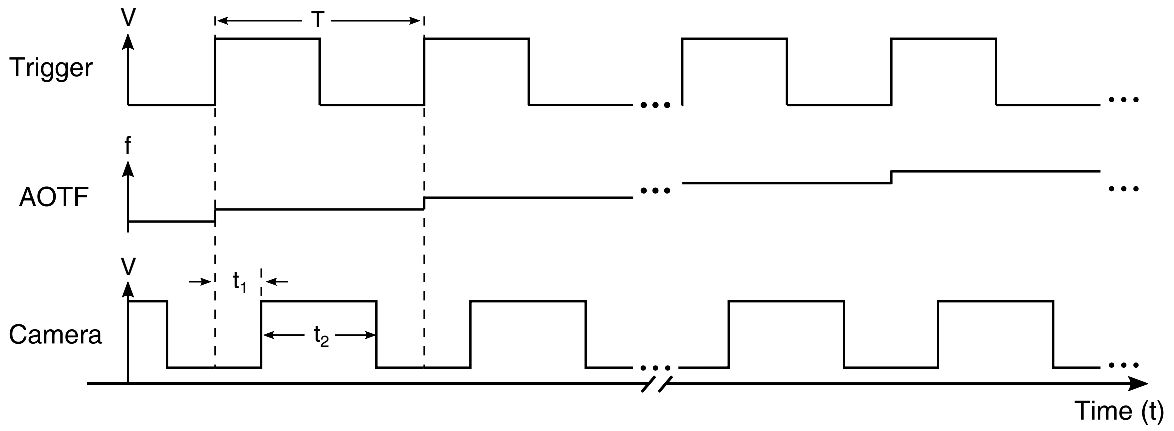

Figure S3: Timing sequences for the involved devices. The trigger signal is a train of square waves with a period of T. At each rising edge, the operation frequency of the RF signal for the AOTF is increased with a user-defined step, which allows to scan the central wavelength of the filter. The camera is operated with a temporal delay t_1 and an exposure time t_2 during each period.

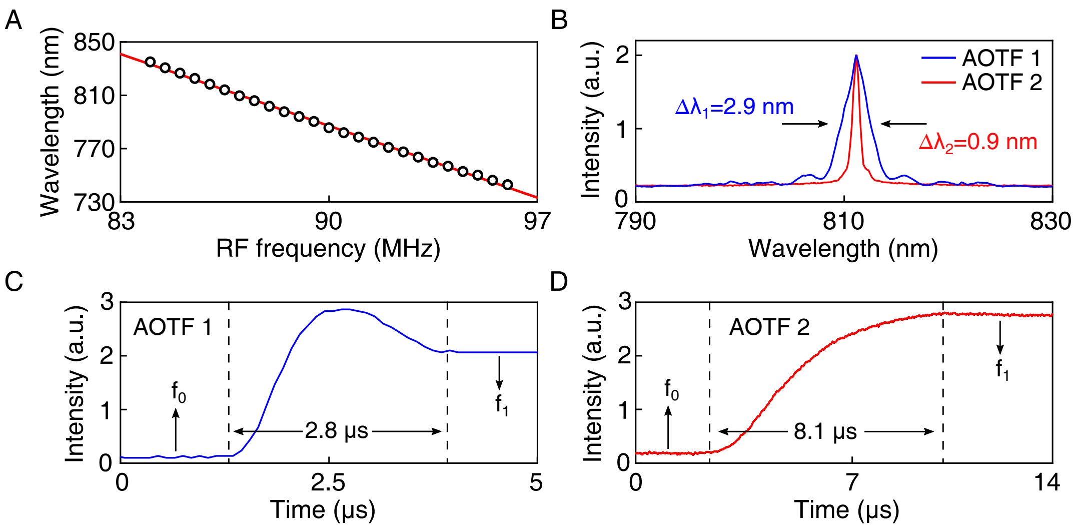

Figure S4: Performance characterization of the AOTF. (A) Central wavelength of the filter has a linear dependence on the driving frequency of the RF signal. (B) Typical filtered spectra for two AOTFs, which indicate wavelength bandwidths of 2.9 and 0.9 nm. (C,D) Temporal responses of the two AOTFs during the wavelength switching, which show completion time of 2.8 and 8.1 μs.

resolution is measured to be 2.9 and 0.9 nm for two AOTFs with different crystal lengths. The use of a longer crystal imposes a more strict phase-matching condition for the photon-phonon interaction, thus resulting in a narrower bandpass [6]. The associated penalty is a slower response speed as evaluated by the temporal traces shown in Figs. S4(C) and (D). The duration for a complete spectral transition is measured to be about 2.8 and 8.1 μs for AOTF1 and AOTF2, respectively. In both cases, the achieved spectral switching time below 10 μs is much smaller than the typical exposure time of 100 μs set for the camera. In contrast the previous AOTF-based MIR spectral imaging [7, 8], the upconversion approach allows to use a visible-band AOTF with a higher performance and a lower cost. Notably, the commercial AOTFs beyond 4.5 μm are currently not available. Therefore, the presented method for the MIR upconversion hyperspectral imaging would be attractive for long-infrared wavelengths or terahertz frequencies, where fast high-definition cameras and agile spectral filters are typically hard to access.

Supplementary Note 4: Spatial resolution of imaging system

As illustrated in Fig. S1(A), the spatial resolution of the upconversion imaging system is determined by the pump size or the crystal aperture, whichever is smaller [2, 4]. At the presence of a Gaussian pump, only Fourier components at low spatial frequencies can be efficiently converted. The low-pass filtering effect is commonly observed in the 4-f imaging system. More rigorously, the upconverted image under a coherent illumination can be expressed as [4, 9]:

$$I_u(x', y') \propto \left| E_{\text{object}}\left(-\frac{\lambda_s f_1}{\lambda_u f_2}x', -\frac{\lambda_s f_1}{\lambda_u f_2}y'\right) \otimes \left[\frac{\pi w_p^2}{(\lambda_u f_2)^2} e^{-\frac{(x'^2+y'^2)\pi^2 w_p^2}{(\lambda_u f_2)^2}}\right]\right|^2, \tag{6}$$

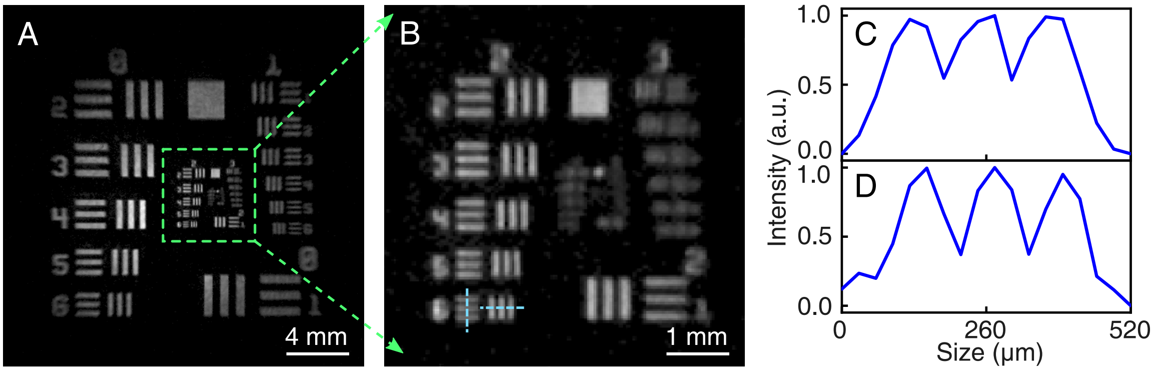

Figure S5: Characterization of the spatial resolution for the MIR upconversion imaging system. (A) Recorded monochromatic image at 2.8 μm for a USAF-1951 resolution target. (B) Zoom-in of the central part in the test chart. The elements 6 in group 2 are denoted by the dashed lines. The bar width is specified to be 70 μm. (C,D) Corresponding cross sections along the vertical and horizontal directions indicated by the blue dashed lines in (B).

where w_p denotes the pump radius defined by the half-width at $1/e^2$ intensity. The Gaussian pump serves as the point spread function of the imaging system, which determines the spatial resolution through the convolution operation. The resultant resolution at the object plane is given by [4]:

$$\mathcal{R} = \frac{2 f_1 \lambda_s}{\pi w_p} \ . \tag{7}$$

In our experiment, the pump diameter is engineered to be about 3 mm for adapting the transverse dimensions of the crystal with a thickness of 2 mm and a width of 3 mm. In this case, the pump can be regarded to be a flat-top beam in the vertical direction. The spatial resolution can be modified to be $\mathcal{R} = f_1\lambda_s/D$ [4], where D is the crystal thickness. The vertical resolution is thus calculated be to 69 μm. The spatial resolution is characterized by using a USAF-1951 resolution target. Figure S5(A) presents the recorded image at 2.8 μm, where bars in element 6 of group 2 can be clearly resolved to validate a spatial resolution of 70 μm. The vertical and horizontal cross sections are given in Figs. S5(C) and (D), respectively. The better resolving performance in the horizontal direction is expected from the rectangular aperture of the crystal. The achieved field of view is about one inch in diameter defined by the aperture of the optical lens, thus leading to $\sim 10^5$ resolvable spatial elements. The spatial resolution can be further enhanced by using a wider-aperture crystal and an enlarged pump beam [1, 4].

Supplementary Note 5: Rescaling operation for spectral images

The combination of the snapshot filtering operation and wide-field detection allows for on-screen visualization of monochromatic images. The recorded spectral images are associated with various enlargement factors as discussed previously, which can be corrected with a simple rescaling operation for obtaining the spectral data cube. In our experiment, the center wavelength of the filter is precisely inferred from the set frequency of the RF signal onto the AOTF based on the

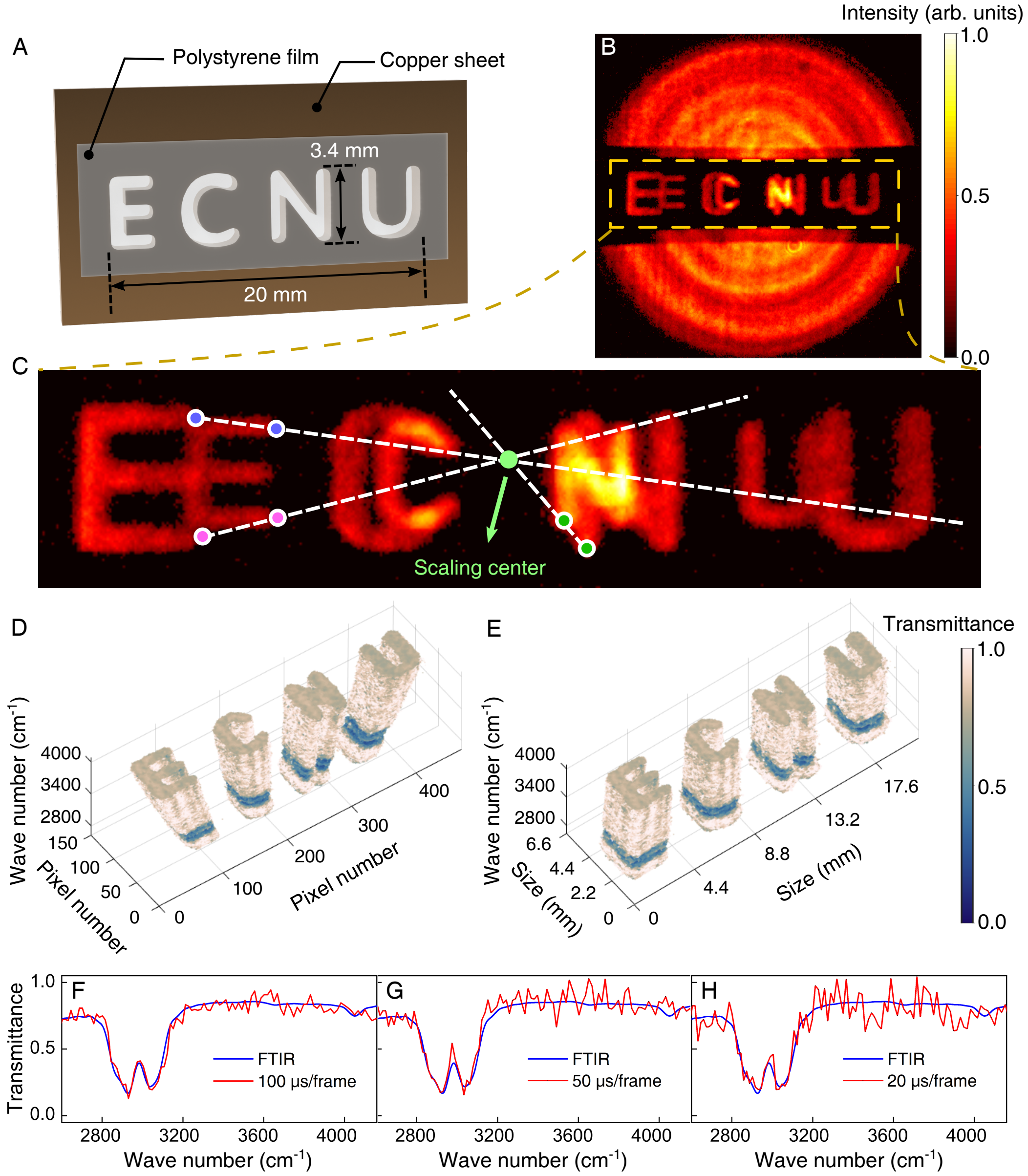

Figure S6: Rescaling operation for the upconversion spectral images. (A) Tested sample with a polystyrene film, which is placed on a copper sheet engraved with four letters. (B) Recorded images for two spectral channels at 2645 and 4010 cm^{-1}. (C) Zoomed-in illustration for the central part relevant to the copper sheet. A spatial enlargement can be observed due to the wavelength-dependent scaling factors, which is used to identify the scaling center. (D) Raw data for the spectral images from 2600 to 4160 cm^{-1}. (E) Corrected hyperspectral images. (F-H) Measured absorption spectrum for the polystyrene film in an exposure time of 100 μs (F), 50 μs (F), and 20 μs (F), respectively.

calibrated curve in Fig. S4(A). Subsequently, the corresponding spatial scaling factor is determined from Eq. (4). Since the scaling operates in the radial direction, it is imperative to identify the scaling center for the image correction.

To this end, a test sample with a pre-determined spatial structure is prepared by placing a

polystyrene film onto a copper sheet engraved with an acronym, as illustrated in Fig. S6(A). Figure S6(B) presents the recorded upconversion image with two superposed spectral components at 2645 and 4010 cm^{-1}. The displayed rings may stem from the phase-matching condition for the nonlinear upconversion, where a slight variation of conversion efficiency is presented along the radial direction. This effect can be removed by subtracting the signal image with a reference that is acquired without the presence of samples. The relevant portion for the sheet copper is enlarged as given in Fig. S6(C), which clearly shows a radial offset for the dual-color patterns. In the recognition of several featured positions on the letters, the corresponding expanding direction can be identified by drawing a line through the two particular points. The intersection point among these lines thus defines the origin of the radial enlargement. After the correction of the scaling factor, the actual size of each monochromatic images can be obtained. Figures S6(D) and (E) present the spectral data cubes within a spectral coverage of 2600-4160 cm^{-1} without and with the rescaling operation, respectively. The absorption spectrum at a specific spatial coordinate within the letters is plotted in Figs. S6(F-H) in the case of different exposure time for the camera from 100 to 20 μs. As expected, a faster spectral imaging speed can be realized at the expense of an reduced signal-to-noise ratio (SNR). Additionally, limited by the speed of readout electronics, the total number of involved pixels will decrease at a higher frame rate. In our experiment, the exposure time is typically set to be 100 μs with an aim to ensure high SNRs and high definition of the acquired spectral images. The frame rate for the monochromatic imaging corresponds to 10 kHz, which represents a record-high speed for MIR hyperspectral imaging with a large spatial format of 0.5 megapixels.

Supplementary Note 6: Algorithm for space-multiplexed spectral imaging

Multiplexed imaging provides an effective approach to boost the acquisition speed, where multiple images can be captured simultaneously within a single exposure [10]. To this end, the AOTF is operated in the parallel filtering modality, which allows us to facilitate multi-spectral imaging at several user-defined discrete wavelengths. In our imaging architecture, there exists a radial blurring in the polychromatic upconverted image, which is ascribed to the phase-matching requirement in the transverse direction. It is the blurring effect that gives rise to an effective spatial dispersion under the broadband illumination. Consequently, different spectral components will be mapped into different spatial positions according to the wavelength-dependent scaling factors.

Generally, each pixel in the recorded snapshot contains the information of many spectral bands. It is indeed a non-trivial task to extract contributions at different wavelengths. In the following proof-of-principle demonstration, a very simple scenario is considered to illustrate the basic idea of MIR space-multiplexed spectral imaging. Here, we assume that the sample is homogenous and is covered by an engraved mask with binary transmissions. In this case, the spectral and morphological profile can be extracted by using an iterative algorithm as presented below.

- ***Algorithm model and initial parameters***

In the case of n spectral channels filtered by AOTF, the intensity distribution of the recorded

snapshot image is given by

$$I(x,y) = \sum_{i=1}^{i=n} I^{(\lambda_i)}(x,y) = \sum_{i=1}^{i=n} T_i \cdot t_i(x,y) \times I_0^{(\lambda_i)}(x,y) \ , \tag{8}$$

where $I^{(\lambda_i)}(x,y)$ and $I_0^{(\lambda_i)}(x,y)$ are the monochromatic images intensity with and without the presence of the sample, and T_i denotes the transmittance at a spectral channel of λ_i, $t_i(x,y)$ is the spatial position map of the sample. For each monochromatic image, the transmission at the location (x,y) where the sample exists is defined as $t_i(x,y) = 1$, otherwise $t_i(x,y) = 0$. As shown in Fig. S7(A), the boundary for each spectral image can be identified by recognizing the particular shape the target. The farthest rim with a radius of L_1 corresponds to the smallest wavelength, which can be identified from the furthest effective signal (with an intensity value larger than a user-defined background threshold) in the captured image. Then, the radius L_i of the inner boundary

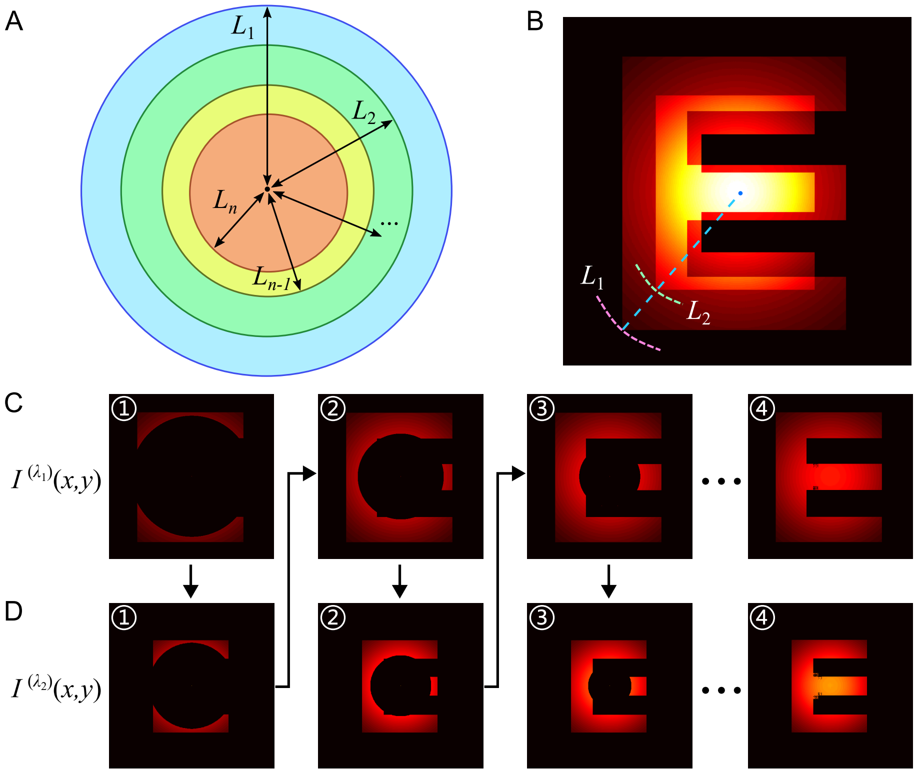

Figure S7: Decomposition process for the snapshot multi-spectral image. (A) Schematic diagram of solving regions. The radius for each boundary is determined by the scaling factor at each spectral channel. (B) Simulated snapshot with the superposition of spectral images for the letter "E". Two spectral channels ($\lambda_1 < \lambda_2$) are illustrated here for the sake of simplicity. The boundaries can be identified from the featured points. The annulus between the boundaries L_1 and L_2 contains only the imaging information at λ_1, while the inward region from the boundary L_2 is overlaid with dual-color spatial information. (C,D) Iterative steps for solving the two monochromatic images at λ_1 (C) and λ_2 (D), respectively.

for each wavelength can be given by

$$L_i = L_1 \times \frac{M_i}{M_1} \ , \tag{9}$$

where the enlargement factor M_i is determined by Eq. (4). Moreover, the scaling factor determines the relationship between the position maps $t_i(x,y)$ and $t_k(x,y)$ at two different wavelengths as:

$$t_i(x,y) = t_k(\frac{M_k}{M_i}x, \frac{M_k}{M_i}y) \ , \tag{10}$$

where we define the scaling center at the origin. For example, Fig. S7(B) illustrates the boundaries of a dual-wavelength spectral image for a transmission mask engraved with a letter "E". For the region between the boundaries L_1 and L_2, there is only the spectral imaging information at the wavelength λ_1. For inward zones, more than one spectral contributions should be taken into account. Therefore, the key to this algorithm lies in solving the spatial distribution of sample and the transmittance at each wavelength.

- ***Determination of annulus region to be calculated***

In the proposed algorithm, we iteratively obtain the information in each annulus from outside to inside. We start from the annulus region between L_1 and L_2, *i.e.*, $L_2 < \sqrt{x^2+y^2} \leq L_1$, where there is only the spectral imaging information at the wavelength λ_1. In this region, if a pixel (x,y) is an effective signal, then we have $t_1(x,y) = 1$. As shown in Fig. S7(C1), T_1 can be simply obtained from $T_1 = \frac{I(x,y)}{I_0^{(\lambda_1)}(x,y)}$. Meanwhile, according to the scaling rule, there will be signal pixels at $(\frac{M_2}{M_1}x, \frac{M_2}{M_1}y)$ related to the wavelength λ_2, *i.e.*, $t_2(\frac{M_2}{M_1}x, \frac{M_2}{M_1}y) = t_1(x,y) = 1$. Hence, the spatial information for $t_2(x,y)$ can be determined within the range $\frac{M_2}{M_1}L_2 < \sqrt{x^2+y^2} \leq L_2$. Meanwhile,

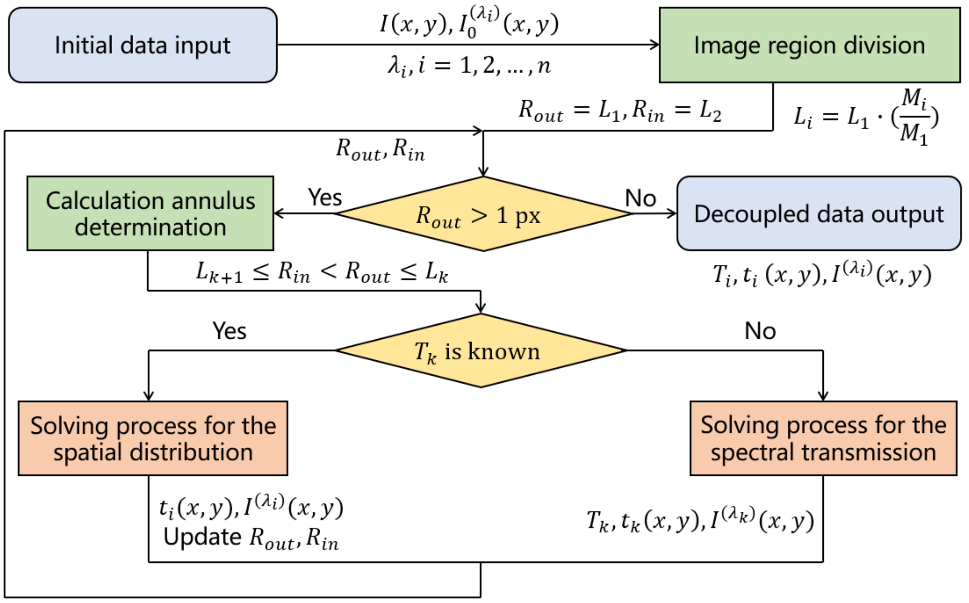

Figure S8: Block diagram of the process for decoupling the snapshot spectral image. Each block is a subroutine and the arrow indicates the direction of the data flow.

the spectral transmittance T_2 needs to be resolved, as well as the spatial information for $t_1(x, y)$ in this region. To this end, two solving processes are used as detailed below to infer the spectral and spatial information, respectively. We can repeat the operation for an annulus region defined by previously calculated area based on the scaling rule. Finally, monochromatic images at each spectral channel can be reconstructed after multiple iterations, as illustrated by the processing flows in Figs. S7(C) and (D). Below, we provide more details on the reconstruction process that has been extended to a more general case with more spectral channels.

Figure S8 presents the flow diagram of the reconstruction algorithm. In the algorithm, it is important to define a suitable annulus region at each iteration step. We initiate the value of each unknown parameter to be 0, *i.e.*, $t_i(x, y) = 0$ and $T_i = 0$, and define the outer radius of each calculated annulus region as R_{out} and the inner radius as R_{in}, *i.e.*, $R_{in} < \sqrt{x^2 + y^2} \leq R_{out}$. Its initial value is $R_{out} = L_1$ and $R_{in} = L_2$. First, we need to identify two adjacent boundaries, between which the annulus to be calculated is located, *i.e.*, $L_{k+1} \leq R_{in} < R_{out} \leq L_k$. The significance of this step is to ensure that the image information in this calculation area is jointly contributed by wavelengths from λ_1 to λ_k. We check whether the spectral transmittance T_k of the region is known. If not, we proceed with the ***Solving process for the spectral transmission*** to get T_k. Otherwise, the ***Solving process for the spatial distribution*** will be executed to obtain spatial maps from $t_1(x, y)$ to $t_k(x, y)$ and intensity distributions from $I^{(\lambda_1)}(x, y)$ to $I^{(\lambda_k)}(x, y)$ in this annulus region. After the two previous steps, all the information within the region has been calculated. Then, we can update the radius for the next annulus region. Specifically, the outer and

---

Algorithm - Solving process for the spectral transmission

---

input: $I(x, y)$, k, $T = [\,]$, $t_i(x, y)$, $I^{(\lambda_i)}(x, y)$, $I_0^{(\lambda_i)}(x, y)$, R_{in}, R_{out}
if $k = 1$
  for x, y satisfy the condition $[I(x, y) > K \;\&\&\; R_{in} < \sqrt{x^2 + y^2} \leq R_{out}]$
    $t_1(x, y) = 1$, $T = [T,\ \frac{I(x,y)}{I_0^{(\lambda_1)}(x,y)}]$, $I^{(\lambda_1)}(x, y) = I(x, y)$
  end for
  $T_1 = \mathrm{mean}(T)$
else if $k \neq 1$
  for x, y in X, Y && $t_1(x, y) = 1$
    $t_k(\frac{M_k}{M_1}x, \frac{M_k}{M_1}y) = 1$
  end for
  for x, y satisfy the condition $[R_{in} < \sqrt{x^2 + y^2} \leq R_{out} \;\&\&\; t_k(x, y) = 1]$
    $T = [T,\ \frac{I(x,y) - \sum_{i=2}^{i=n} I^{(\lambda_i)}(x,y)}{I_0^{(\lambda_k)}(x,y)}]$, $T = [T,\ \frac{I(x,y) - T_1 \cdot I_0^{(\lambda_1)}(x,y) - \sum_{i=2}^{i=n} I^{(\lambda_i)}(x,y)}{I_0^{(\lambda_k)}(x,y)}]$
  end for
  $T_k = \mathrm{mode}(T)$, $I^{(\lambda_k)}(x, y) = T_k \cdot t_k(x, y) \cdot I_0^{(\lambda_k)}(x, y)$
end if
output: T_k, $t_k(x, y)$, $I^{(\lambda_k)}(x, y)$

---

inner radius become:

$$R_{out} = R_{in},\ R_{in} = \begin{cases} \max(L_{k+1}, \frac{M_2}{M_1}R_{in}), \text{ if } R_{in} > L_{k+1}, \\ \frac{M_2}{M_1}R_{in}, \text{ if } R_{in} = L_{k+1}. \end{cases} \tag{11}$$

It is worth noting that for $R_{out} \leq L_n$ the updating rule at each iteration step should be modified as $R_{out} = R_{in}, R_{in} = \frac{M_2}{M_1}R_{in}$.

- ***Solving process for the spectral transmission***

The calculation process for transmittance T_k is shown in the pseudo-code below, where K is the threshold used to filter out noise and X, Y are the coordinate ranges of the entire image. For $k = 1$, the region to be calculated satisfies $L_2 < \sqrt{x^2 + y^2} \leq L_1$. In this case, there is only one involved wavelength. The relevant calculation has been described previously. For the case $k \geq 2$, more spectral channels should be taken into account. We elaborate the reconstruction process with an example for $k = 2$. The spatial map $t_2(x, y)$ in the annulus region $R_{in} < \sqrt{x^2 + y^2} \leq R_{out}$ can be derived from previously calculated region in $t_1(x, y)$. For the spatial map where $t_2(x, y) = 1$, we have $T_2 = \frac{I(x,y) - T_1 \cdot I_0^{(\lambda_1)}(x,y)}{I_0^{(\lambda_2)}(x,y)}$ under the assumption of $t_1(x, y) = 1$, or $T_2 = \frac{I(x,y)}{I_0^{(\lambda_2)}(x,y)}$ under the assumption of $t_1(x, y) = 0$. The true value of the transmittance T_2 can be identified from the mode number (the most frequent value in an array) for all the calculated pixels. This method is also applicable for $k \geq 3$. Within the updated calculation region, the spatial maps from $t_2(x, y)$ to $t_k(x, y)$ can be obtained via the scaling operation on the previously calculated region in $t_1(x, y)$. Similarly, we need to determine the $t_1(x, y)$ in the current calculation region, and the transmittance T_k. The contributions from other spectral components at pixels where $t_k(x, y) = 1$ can be obtained with $I^{(\lambda_i)}(x, y) = T_i \cdot t_i(x, y) \times I_0^{(\lambda_i)}(x, y),\ i = 2, ..., k - 1$. Therefore, it is possible to infer the T_k via the mode-searching algorithm.

- ***Solving process for the spatial distribution***

During the reconstruction process, if the transmittances from T_1 to T_k of the sample have been obtained, the spatial intensity distributions from $I^{(\lambda_1)}(x, y)$ to $I^{(\lambda_k)}(x, y)$ can be calculated by

Algorithm - Solving process for the spatial distribution
input: $I(x, y)$, k, $t_i(x, y)$, $I^{(\lambda_i)}(x, y)$, $I_0^{(\lambda_i)}(x, y)$, R_{in}, R_{out}
for x, y satisfy the condition $[I(x, y) > K$ && $\sqrt{x^2 + y^2} > R_{in}]$
$I^{(\lambda_1)}(x, y) = I(x, y) - \sum_{i=2}^{i=n} I^{(\lambda_i)}(x, y)$, update $t_1(x, y)$
end for
for x, y in X, Y && $t_1(x, y) = 1$
for $i = 2 : n$
$t_i(\frac{M_i}{M_1}x, \frac{M_i}{M_1}y) = 1, I^{(\lambda_i)}(\frac{M_i}{M_1}x, \frac{M_i}{M_1}y) = T_i \cdot I_0^{(\lambda_i)}(\frac{M_i}{M_1}x, \frac{M_i}{M_1}y)$
end for
end for
update R_{in}, R_{out}
output: $t_i(x, y)$, $I^{(\lambda_i)}(x, y)$, R_{in}, R_{out}

combining the spatial map $t_1(x,y)$ in the previous calculation area. In this case, there is only $t_1(x,y)$ unknown in the selected region $\sqrt{x^2+y^2} > R_{in}$. The value of $t_i(x,y)$(where $i \geq 2$) is obtained based on the scaled spatial map $t_1(x,y)$ by the factor M_i/M_1 in the previous calculation region $\sqrt{x^2+y^2} \geq R_{out}$. Therefore, the value $I^{(\lambda_1)}(x,y)$ in this area can be determined by subtracting the spectral intensity $\sum_{i=2}^{i=n} I^{(\lambda_i)}(x,y)$ from the snapshot image $I(x,y)$. Once the $I^{(\lambda_1)}(x,y)$ of the region is obtained, the updated value $t_1(x,y)$ can be used to repetitively calculate the spatial map and spectral intensity for other spectral channel in the next annular region.

As a proof of principle for the snapshot MIR spectral imaging, a test scene is prepared by placing a polystyrene onto a copper sheet with four engraved letters as shown in Fig. S9(A). The spectral image with six wavelength channels is recorded by the CMOS camera with an exposure time of 100 μs. Figures S9(B-G) present the reconstruction results for the corresponding monochromatic images at at 2610, 2827, 3006, 3210, 3444, and 3713 cm^{-1}, respectively. Here, the wavelengths are chosen to be well separated, which leads to a more pronounced blurring effect. The larger radial shift for various spectral components favors the image reconstruction. In general, the spectral

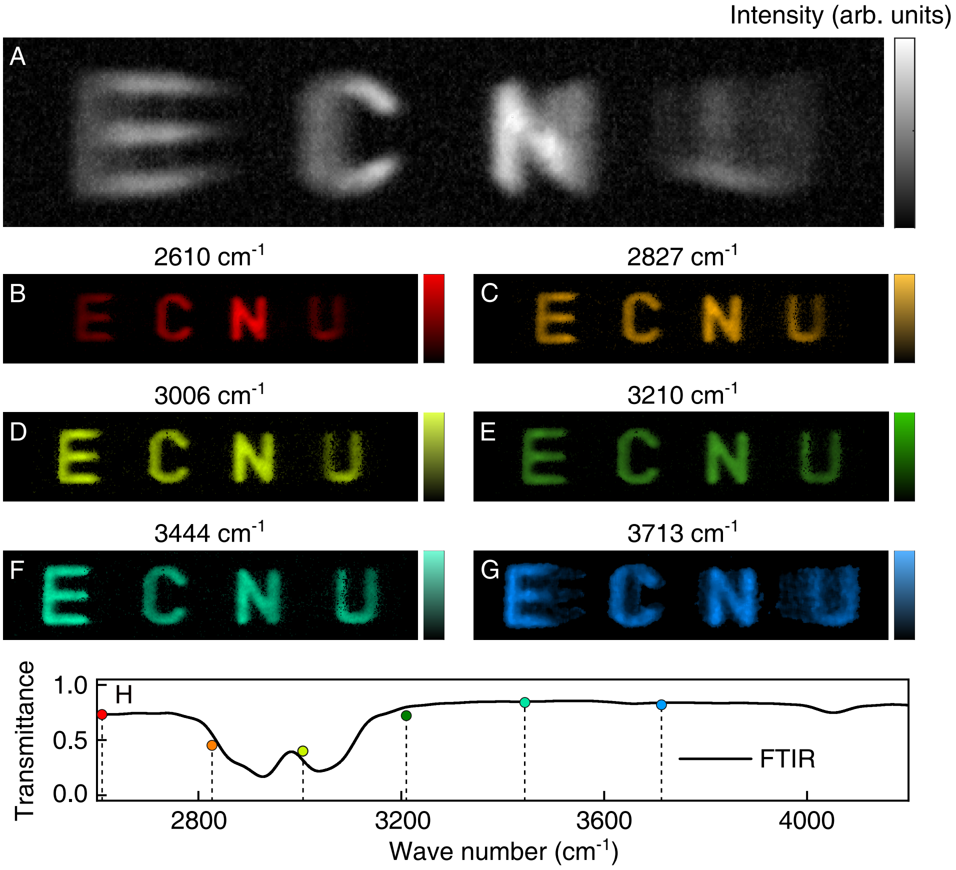

Figure S9: Monochromatic decomposition for the MIR space-multiplexed spectral imaging. (A) Recorded snapshot with the superposition of six spectral images. (B-G) Reconstructed monochromatic image for the spectral band at 2610, 2827, 3006, 3210, 3444, and 3713 cm^{-1}, respectively. Note that these decomposed images can be spatially corrected by a simple rescaling operation as detailed in Supplementary Note 5. (H) Measured absorptions at the corresponding wavelengths, which are consistent to the FTIR reference.

and spatial information is coupled together, and the extraction of a multi-spectral cube from the input dispersed gray image is usually complicated in such an ill-posed optimization problem. The assistance with more advanced algorithms may improve the reconstruction quality and applicability, for instance, resorting to computation spectral imaging techniques based deep learning [11].

Supplementary Note 7: Comparison of wide-field MIR spectral imagers

So far, there have been various approaches established for the implementation of wide-field MIR hyperspectral imaging, which can be divided into two categories based on direct and indirect infrared detection. The performance comparison is summarized in Table S1. In the direct method, infrared FPAs based on microbolometers or narrow-bandgap semiconductors are typically used to facilitate the wide-field imaging. For instance, the integration of a multi-pixel detector in Fourier transform infrared (FTIR) imaging spectrometers can significantly boost the data acquisition speed [16, 17], with a comparison to the time-consuming operation based on point scanning. However, the FTIR technique typically necessitates an interferometric scan plus subsequent processing to reconstruct the MIR absorption spectrum, which can slow down the overall acquisition process. Alternatively, quantum cascade lasers (QCLs) have been used for MIR spectral imaging due to the high illumination brightness, high spectral resolution, and fast wavelength tunability [18–20]. Moreover, MIR spectral imaging over an octave-spanning spectrum can be realized by using multiple synchronized QCLs [19]. Recently, a broadband supercontinuum (SC) laser source is leveraged to implement the MIR hyperspectral imaging with the help of an AOTF [21]. In all above direct approaches, the performance is currently limited by the infrared imagers that have long been plagued with high dark noise, low pixel count, and limited frame rate. Specifically, the frame rate for 640×512 is restricted to be below 100 Hz [21]. A faster readout rate up to kHz is only possible at the cost of greatly reduced number of pixels, *e.g.*, a spatial format of 128×128 pixels [18]. Moreover, the MIR imagers usually need a cryogenic operation to suppress the severe thermal noise for improving the detection sensitivity.

In the context, indirect approaches have been developed to address the aforementioned limitations of arrayed MIR detectors, where the information encoded in the MIR region is transferred to the visible or near-infrared range for leveraging more mature large-bandgap semiconductor technology. Such a frequency conversion step can be realized in various ways. For instance, MIR photothermal imaging (PTI) is implemented by using a visible laser to probe the infrared absorption-induced thermal lensing effect in the sample [15]. In the wide-field PTI, the required intensive MIR illumination usually resorts to an optical parametric oscillator (OPO) with high pulse energy. The spectral imaging speed is limited by the slow spectral tuning speed of the OPO, especially to cover a wide spectrum. Notably, the use of QCLs in the MIR photothermal imaging allows to access fast-tuning illumination sources over a wide spectral range. In combination with a fast and sensitive camera, the advent of high-power QCLs at high repetition rate would make it possible to implement the wide-field imaging modality to significantly increase the frame rate. Another attractive conversion method exploits the intrinsic optical nonlinearity of the detector material itself based on non-degenerate two-photon absorption (NTA). Recently, a NTA-based spectral imaging

is implemented with a high-definition InGaAs camera [14]. The spectral scanning rate can be fast during a single sweep, yet the dwell time between sequential sweeps is relatively longer due to the mechanical inertial of the involved translational stage. As a result, the demonstrated refreshing rate for the datacube acquisition is limited to Hz level at the continuous operation modality. Additionally, the spectral coverage is limited to about 530 cm^{-1}, as determined by the spectral width of the OPO source. It is worth mentioning that the refreshing rate in the NTA-based scheme could be substantially improved by using a voice coil actuator as the delay line, which would facilitate a real-time hyperspectral imaging at high definition.

Another conversion method exploits the parametric interaction within a nonlinear crystal, which has been proved to be the most efficient way for spectral transduction based on the second-order SFG process [4]. In the presence of long nonlinear crystals, the phase-matching requirement generally leads to a narrow acceptance window for the infrared signal in both the spectral and spatial domains. A wide-field spectral upconversion imaging is typically realized based on scanning phase-matching parameters, such as operation temperature or crystal orientation [12], which inevitably limits the spectral imaging speed. Very recently, a broadband upconversion imaging is

Table S1: Performance comparison of wide-field MIR hyperspectral imaging systems.

Type	Ref.	Source	Range (cm^{-1})	Res. (cm^{-1})	Band Num.	Pixel Num.	Time (per cube)	Power (/nm/cm^2)	Detector
SFG	This	SC	2600-4085	16/50$^{(a)}$	100	1024 × 1024	16 ms$^{(b)}$	7 μW	Si
						768 × 768	10 ms		
	[12]	OPO	2500-4348	3.6-7.6	62	658 × 496	25 s$^{(c)}$	20 mW	Si
	[13]	DFG	640-3015	2.6-3.7	1069	640 × 480	8 s	1 μW	Si
NTA	[14]	OPO	2735-3265	8.4	63	1280 × 1024	1.1 s	17 μW	InGaAs
PTI	[15]	OPO	2830-2950	10	13	2016 × 2016	260 ms	4 W	Si
Direct	[16]	Globar	988-1950	16	FTIR$^{(d)}$	96 × 96	93 ms	/$^{(e)}$	MCT
	[17]	Globar	1850-6667	1	FTIR$^{(d)}$	256 × 160	60 s	/$^{(e)}$	InSb
	[18]	QCL	952-1351	2	137	128 × 128	95 ms	18 μW	MCT
	[19]	QCL	777-1904	2	282	128 × 128	120 s	121 mW	MCT
	[20]	QCL	1160-1320	1	565	640 × 480	11.3 s	75 mW	Bolometer
	[21]	SC	2222-5000	1-5	100	640 × 512	1.2 s	7 mW	InSb

(a) The MIR spectral resolutions are corresponding to the filter bandwidths of 0.9 and 2.9 nm for the two AOTFs used in our experiment.

(b) The acquisition time is determined by the maximum frame rate of 6.4 kHz for the full spatial format of the CMOS camera used in our experiment.

(c) Although a wide-field image at a single wavelength can be recorded in 2.5 ms, the exposure time of the camera is set to be 400 ms for improving the image quality in the spectral imaging demonstration.

(d) The spectral imaging is realized based on the FTIR technique.

(e) The illumination power for the used thermal source is not given in these works.

demonstrated based on a thin crystal with a length shorter than 5 μm [13]. The broadband MIR source is prepared by using four-wave difference frequency generation (DFG) based on a high-power Ti:Sapphire laser. An extremely-high pump intensity is required to obtain a pronounced conversion efficiency due to the very short interaction length, which is provided by a complex solid-state laser system. The involved high-energy pulses with ∼mJ operate at a relatively low repetition rate at ∼ kHz, which limits the monochromatic imaging speed. Moreover, the filtering operation for the upconversion images is conducted by rotating a dispersive grating, which thus imposes a stringent limitation on the spectral imaging speed.

In our work, we devise and implement a MIR spectral upconversion imaging at high definition and high speed based on collective innovations in bright supercontinuum illumination, broadband frequency upconversion, rapid acousto-optic filtering, and fast wide-field detection. Here, the full infrared information in spatial and spectral domains is completely transferred into the visible upconversion replica in a single shot, which contrasts to previous works that require parameter scanning and post-processing. The achieved performances here have significantly surpassed the reported benchmarks among various MIR spectral imaging techniques, which allows us to address the long-standing quest to realize video-rate acquisition of MIR hyperspectral data cubes with large spatial formats and broad spectral channels.

Notably, it is important to control the illumination intensity in biochemical imaging. Here the illumination strength is about 7 μW/nm/cm^2, which is comparable to the values in other spectral imaging systems. The illumination power could further be reduced by improving the conversion efficiency, or resorting to the spatial multiplexing scheme. To suppress the spatial coherence of the laser source, a rotatory diffuser can be used to smooth the speckles [12]. Therefore, the achieved features of high acquisition rate, wide-field operation, and broadband spectral coverage would now open up new possibilities in high-throughput characterization of dynamic processes in chemical, medical, and bio-related fields.

Supplementary Video

Supplementary Video 1: High-speed MIR spectral videography for capturing the liquid injection process. Each monochromatic image is recorded in 0.1 ms, corresponding to a frame rate of 10 kHz. The total acquisition time for 100 spectral bands is 10 ms over a spectral coverage from 2600 to 4085 cm^{-1}.

Supplementary Video 2: Wide-field MIR hyperspectral imaging for real-time visualization of the liquid mixing dynamics. The chemical contrast is manifested by the overlay of the red and green frames that are corresponding to two specific spectral channels at 3050 and 3350 cm^{-1}. The refreshing rate for the data-cube sequences reaches to 100 kHz.

[1] K. Huang, J. Fang, M. Yan, E Wu, and H. Zeng, "Wide-field mid-infrared single-photon upconversion imaging," *Nat. Commun.* **13**, 1077 (2022).

[2] Y. Wang, J. Fang, T. Zheng, Y. Liang, Q. Hao, E. Wu, M. Yan, K. Huang, and H. Zeng, "Mid-infrared single-photon edge enhanced imaging based on nonlinear vortex filtering," *Laser Photonics Rev.* **15**, 2100189 (2021).

[3] H. Zhang, S. Kumar, Y. M. Sua, S. Zhu, and Y.-P. Huang, "Near-infrared 3D imaging with upconversion detection," *Photonics Res.* **10**, 2760-2767 (2022).

[4] A. Barh, P. J. Rodrigo, L. Meng, C. Pedersen, and P. Tidemand-Lichtenberg, "Parametric upconversion imaging and its applications," *Adv. Opt. Photonics* **11**, 952-1019 (2019).

[5] Y. Pang, K. Zhang, and L. Lang, "Review of acousto-optic spectral systems and applications," *Front. Phys.* **10**, 1102996 (2022).

[6] L. Bei, G. I. Dennis, H. M. Miller, T. W. Spaine, and J. W. Carnahan, "Acousto-optic tunable filters: fundamentals and applications as applied to chemical analysis techniques," *Prog. in Quant. Electron.* **28**, 67-87 (2004).

[7] J. D. Ward, S. Valle, C. Pannell, N. P. Johnson, "Acousto-Optic Tunable Filters (AOTFs) Optimised for Operation in the 2-4μm region," *J. Phys. Conf. Ser.* **619**, 012054 (2015).

[8] H. Zhao, Z. Ji, G. Jia, Y. Zhang, Y. Li, and D. Wang, "MWIR thermal imaging spectrometer based on the acousto-optic tunable filter," *Appl. Opt.* **56**, 7269 (2017).

[9] C. Pedersen, E. Karamehmedović, J. S. Dam, and P. Tidemand-Lichtenberg, "Enhanced 2D-image upconversion using solid-state lasers.," *Opt. Express* **17**, 20885-20890 (2009).

[10] X. Hua, Y. Wang, S. Wang, X. Zou, Y. Zhou, L. Li, F. Yan, X. Cao, S. Xiao, D. P. Tsai, J. Han, Z. Wang, S. Zhu, Ultra-compact snapshot spectral light-field imaging. *Nat. Commun.* **13**, 2732 (2022).

[11] L. Huang, R. Luo, X. Liu, X. Hao, Spectral imaging with deep learning. *Light Sci. Appl.* **11**, 61 (2022).

[12] S. Junaid, S. C. Kumar, M. Mathez, M. Hermes, N. Stone, N. Shepherd, M. Ebrahim-Zadeh, P. Tidemand-Lichtenberg, and C. Pedersen, "Video-rate, mid-infrared hyperspectral upconversion imaging," *Optica* **6**, 702-708 (2019).

[13] Y. Zhao, S. Kusama, Y. Furutani, W.-H. Huang, C.-W. Luo, and T. Fuji, "High-speed scanless entire bandwidth mid-infrared chemical imaging," *Nat. Commun.* **14**, 3929 (2023).

[14] D. Knez, B. W. Toulson, A. Chen, M. H. Ettenberg, H. Nguyen, E. O. Potma, and D. A. Fishman, "Spectral imaging at high definition and high speed in the mid-infrared," *Sci. Adv.* **8**, eade4247 (2022).

[15] T. Yuan, M. A Pleitez, F. Gasparin, V. Ntziachristos, "Wide-Field Mid-Infrared Hyperspectral Imaging by Snapshot Phase Contrast Measurement of Optothermal Excitation," *Analytical chemistry* **93**, 15323-15330 (2021).

[16] K. L. A. Chan, S. G. Kazarian, "FT-IR Spectroscopic Imaging of Reactions in Multiphase Flow in Microfluidic Channels," *Anal. Chem.* **84**, 4052-4056 (2012).

[17] J. Meléndez, G. Guarnizo, "Fast Quantification of Air Pollutants by Mid-Infrared Hyperspectral Imaging and Principal Component Analysis," *Sensors* **21**, 2092 (2021).

[18] D. B. Kelley, A. K. Goyal, N. Zhu, D. A. Wood, T. R. Myers, P. Kotidis, C. Murphy, C. Georgan, G. Raz, R. Maulini, A. Müller, "High-speed mid-infrared hyperspectral imaging using quantum cascade lasers," *Proc. SPIE* **10183**, 1018304 (2017).

[19] K. Yeh, S. Kenkel, J.-N. Liu, R. Bhargava, "Fast Infrared Chemical Imaging with a Quantum Cascade Laser," *Analytical chemistry* **87**, 485 - 493 (2015).

[20] K. Haase, N. Kröger-Lui, A. Pucci, A. Schönhals, W. Petrich, "Real-time mid-infrared imaging of living microorganisms." *J. Biophotonics* **9**, 61-66 (2016).

[21] M. Farries, J. Ward, S. Valle, G. Stephens, P. Moselund, K. V. D. Zanden, and B. Napier, "Mid infrared hyper-spectral imaging with bright super continuum source and fast acousto-optic tuneable filter for cytological applications," *Journal of Physics: Conference Series* **619**, 12032 (2015).
