# Supplementary material for: Wide-field mid-infrared hyperspectral imaging beyond video rate
Source: Nat Commun. 2024 Feb 28;15:1811. doi: 10.1038/s41467-024-46274-z (PMC10902379; doi:10.1038/s41467-024-46274-z)
Supplement: Supplementary file 3 — Description of Additional Supplementary Files [file 41467_2024_46274_MOESM3_ESM.pdf]

Description of Additional Supplementary Files

File Name: Supplementary Movie 1
Description: High-speed MIR spectral videography for capturing the liquid injection process. Each monochromatic image is recorded in 0.1 ms, corresponding to a frame rate of 10 kHz. The total acquisition time for 100 spectral bands is 10 ms over a spectral coverage from 2600 to 4085 cm^{-1}.

File Name: Supplementary Movie 2
Description: Wide-field MIR hyperspectral imaging for real-time visualization of the liquid mixing dynamics. The chemical contrast is manifested by the overlay of the red and green frames that are corresponding to two specific spectral channels at 3050 and 3350 cm^{-1}. The refreshing rate for the data-cube sequences reaches to 100 kHz.
